# Comparative Outcomes of Pars Plana Vitrectomy Versus Combined Vitrectomy With Phacoemulsification in Proliferative Diabetic Retinopathy and Cataract

**DOI:** 10.7759/cureus.109723

**Published:** 2026-05-27

**Authors:** James Lim Wen Siang, Norlina Ramli

**Affiliations:** 1 Ophthalmology, University Malaya Medical Centre, Kuala Lumpur, MYS

**Keywords:** cataract extraction, combined surgery, diabetic retinopathy, phacovitrectomy, vitrectomy

## Abstract

Background

Proliferative diabetic retinopathy (PDR) often requires pars plana vitrectomy (PPV) for complications such as vitreous hemorrhage and tractional retinal detachment. Cataract progression following PPV is common, and the role of combined PPV with cataract extraction (PPVCE) remains debated. This study aimed to compare visual outcomes and post-operative complications between PPV alone and PPVCE in patients with PDR and coexisting cataract.

Methods

This retrospective observational study was conducted at Hospital Sultanah Bahiyah, Kedah, Malaysia, and included diabetic patients with PDR and coexisting cataract who underwent either PPV alone or PPVCE. Pre-operative and post-operative data were collected, including best-corrected visual acuity (BCVA) and post-operative complications. The main outcome measures were a change in BCVA and the incidence of post-operative complications. Comparative statistical analyses were performed between the two groups.

Results

A total of 115 eyes were evaluated (PPV=43; PPVCE=72), with a mean follow-up period of 1.27 years. The PPVCE group was significantly older than the PPV group (49.22 vs 43.30 years, p=0.01). Both groups demonstrated improvement in BCVA, with no significant between-group difference in final BCVA at last follow-up (p=0.44). Significant visual improvement was observed in vitreous hemorrhage (VH) cases compared to tractional retinal detachment (TRD). Notably, 48.8% of patients in the PPV-alone group required subsequent cataract extraction during follow-up. There were no significant differences in complication rates, including ocular hypertension and neovascular glaucoma, between the two groups (p=0.33 and p=0.53, respectively).

Conclusion

PPV and PPVCE provide comparable visual and safety outcomes in patients with PDR and cataracts. However, the high rate of subsequent cataract surgery following PPV alone suggests that a combined approach may reduce the need for re-operations and improve overall patient management in appropriately selected patients.

## Introduction

Currently, 537 million adults worldwide are living with diabetes, and this figure is projected to rise to 643 million by 2030 and 783 million by 2045 [1]. Diabetic retinopathy, a common and specific microvascular complication of diabetes, remains the leading cause of preventable blindness among working-age individuals [2]. Two primary complications of diabetic retinopathy result in vision loss, namely diabetic macular edema (DME) and proliferative diabetic retinopathy (PDR) [3]. Additionally, cataracts are a leading cause of blindness worldwide, with individuals with diabetes being three times more likely to develop them [4].

As diabetic retinopathy progresses to PDR, pars plana vitrectomy (PPV) is often necessary to manage vitreous hemorrhage, fibrovascular proliferation, and retinal traction. This procedure may involve the removal of the vitreous body, peeling off the thickened posterior hyaloid, addressing retinal detachment, and improving retinal ischemia [5]. However, one of the most common complications of PPV is the development of nuclear sclerotic cataracts, which occur in 75%-95% of patients within two years of surgery [6]. The risk factors for cataract formation include older age group, pre-existing nuclear sclerosis, and the intraoperative use of silicone oil or gas [7].

The decision to remove the crystalline lens during vitreous surgery in diabetic patients remains a subject of debate. Post-vitrectomy cataract surgery becomes more challenging due to the loss of vitreous support and the increased fragility of the posterior capsule [8,9]. Given the high likelihood of cataract development after vitrectomy, along with the complexities of cataract surgery post-vitrectomy and the potential to reduce the number of surgeries in patients with significant comorbidities, combined surgery (PPV with phacoemulsification, or PPVCE) has emerged as a viable option [10]. However, some studies suggest that combined surgery may increase the risk of post-operative complications, such as neovascular glaucoma, compared to PPV alone [9].

Considering these uncertainties, we conducted this study to compare PPV alone versus PPVCE in patients with PDR and coexisting cataract. We aimed to evaluate whether combined surgery provides comparable visual and safety outcomes while reducing the need for subsequent cataract extraction. These findings may help guide surgical decision-making and optimize patient management.

## Materials and methods

This study was approved by the Institutional Review Board (National Medical Research Register (NMRR ID: 24-03669-M7C (IIR))). The requirement for informed consent was waived due to the retrospective nature of the study. All procedures were conducted in accordance with the tenets of the Declaration of Helsinki and its later amendments.

This observational analysis involved diabetic patients who underwent either PPV alone or combined PPV with phacoemulsification and intraocular lens implantation (PPVCE) at a tertiary referral hospital from July 2022 to December 2023. The decision to perform PPV alone or combined PPVCE was based on the surgeon's clinical judgment after assessment of lens status, severity of cataract, adequacy of posterior segment visualization, patient age, and the likelihood of cataract progression following vitrectomy. As this was a retrospective study, cataract severity was determined clinically from slit-lamp examination records documented by the treating surgeons. A formal standardized cataract grading system was not routinely used.

All vitrectomies were performed by the same experienced vitreoretinal surgeon (KO). Cataract surgeries were carried out either by this surgeon or another skilled cataract surgeon, ensuring consistency in the operating conditions. Phacoemulsification was performed using a clear corneal incision, while vitrectomy was performed using a 23-gauge vitreous cutter.

The inclusion criteria for the study included patients with a confirmed diagnosis of PDR based on a fundus examination, those who underwent either PPV or PPVCE during the study period, and a minimum follow-up period of six months post-operatively. The exclusion criteria included patients with retinal conditions unrelated to diabetic retinopathy, a history of previous vitrectomy or cataract surgery in the studied eye, inadequate follow-up of fewer than six months, and eyes with significant ocular comorbidities unrelated to diabetic retinopathy, such as uveitis or severe corneal opacity.

Pre-operative clinical data collected included patient demographics, medical history related to diabetes, previous diabetic retinopathy interventions (e.g., panretinal photocoagulation or intravitreal injections), and indications for surgery. The type of tamponade used intraoperatively was recorded. Ophthalmic data were collected pre-operatively and at one-month, three-month, six-month, and final visit intervals. Data included best-corrected visual acuity (BCVA), intraocular pressure (IOP), anterior segment examination (lens status, iris neovascularization), and other post-operative complications.

The primary outcome measure was the logarithm of the minimum angle of resolution (logMAR) for BCVA assessed after PPV or PPVCE. The secondary outcomes included the incidence of ocular complications occurring during the follow-up period.

For visual acuity analyses, only eyes with measurable BCVA were included. Eyes recorded as perception of light (PL) or no perception of light (NPL), either pre-operatively or post-operatively, were excluded from quantitative logMAR analysis because reliable numerical conversion was not possible. However, these eyes were retained in descriptive summaries and post-operative complication analyses. 

Statistical analysis was performed using SPSS Statistics version 27 (IBM Corp., Armonk, NY, USA). Continuous variables were presented as mean±standard deviation and compared between groups using the independent-samples t-test. Within-group comparisons of pre-operative and post-operative BCVA were performed using the paired t-test. Categorical variables were presented as frequencies and percentages and analyzed using Pearson’s chi-square test or Fisher’s exact test where appropriate. A p-value of less than 0.05 was considered statistically significant.

## Results

A total of 481 PPV procedures were performed during the study period. After applying the exclusion criteria, 115 eyes from 115 diabetic patients who underwent either PPV or PPVCE were included in the analysis. Of these patients, 64 (55.7%) were women, and 51 (44.3%) were men. The mean follow-up period was 1.27±0.41 years (range: 0.52-2.04 years). The PPV group comprised 43 eyes, while the PPVCE group included 72 eyes. Baseline demographics and comparisons between the two groups are summarized in Table 1. A significant difference in age was observed, with the PPVCE group being older (mean: 49.22 years) than the PPV group (mean: 43.30 years, p=0.001). The majority of patients in both groups were Malay (88%-90%). No significant differences were noted between the two groups in terms of gender, race, pre-operative visual acuity, hypertension, nephropathy, random blood sugar levels, previous panretinal photocoagulation (PRP) laser treatment, intravitreal anti-VEGF (vascular endothelial growth factor) treatments, anesthesia type, or follow-up duration.

**Table 1:** Baseline Demographics and Clinical Characteristics of PPV and PPVCE Groups *Four eyes had PL vision in PPVCE group and excluded from the visual acuity analysis. PPV: Pars plana vitrectomy; PPVCE: Pars plana vitrectomy with cataract extraction; BCVA: Best-corrected visual acuity; PRP: panretinal photocoagulation; PL: perception to light; RBS: Random blood sugar; n: number; VEGF: vascular endothelial growth factor. Data are presented as mean±standard deviation for continuous variables and frequency (percentage) for categorical variables. Continuous variables were compared using an independent-sample t-test, and categorical variables were analyzed using Chi-square or Fisher’s exact test.

Variable	PPV (n=43)	PPVCE (n=72)	p-value
Age (years)	43.30±9.11	49.22±9.50	0.001
Gender, n (%)			0.23
Male	16 (37.2)	35 (48.6%)	
Female	27 (62.8)	37 (51.4%)	
Race, n (%)			0.63
Malay	38 (88.4%)	65 (90.3%)	
Chinese	2 (4.7%)	3 (4.2%)	
Indian	2 (4.7%)	4 (5.6%)	
Others	1 (2.3%)	0	
Pre-operative BCVA (logMAR)	1.58±0.87	1.57±0.85*	0.98
Hypertension, n (%)	30 (69.8%)	59 (81.9%)	0.13
Nephropathy, n (%)	13 (30.2%)	29 (40.3%)	0.28
RBS (mmol/L)	10.25±3.98	10.51±3.80	0.73
Previous laser PRP, n (%)	37 (86.0%)	61 (84.7%)	0.85
Previous intravitreal anti-VEGF, n (%)	37 (86.0%)	64 (88.9%)	0.65
Type of anesthesia, n (%)			0.98
Local anesthesia	24 (55.8%)	40 (55.6%)	
General anesthesia	19 (44.2%)	32 (44.4%)	
Follow-up duration (years)	1.16±0.40	1.33±0.40	0.25

Indications for vitrectomy

The primary indications for vitrectomy (Table 2) included tractional retinal detachment (TRD), vitreous hemorrhage (VH), and a combination of TRD with VH. In the PPVCE group, TRD was the predominant indication, whereas the PPV group showed a higher incidence of combined TRD and VH. Additionally, VH alone was more common in the PPV group than in the PPVCE group.

**Table 2:** Indications for Vitrectomy in PPV and PPVCE Groups PPV: Pars plana vitrectomy; PPVCE: Pars plana vitrectomy with cataract extraction; TRD: Tractional retinal detachment; VH: Vitreous hemorrhage

Indication	PPV (n=43)	PPVCE (n=72)
TRD	16 (37.2%)	37 (51.4%)
VH	9 (20.9%)	8 (11.1%)
TRD+VH	18 (41.9%)	27 (27.5%)

Type of endotamponades used

The use of endotamponades varied between the PPV and PPVCE groups (Table 3). Silicone oil was the most commonly used tamponade in both groups. However, the PPV group had a higher proportion of cases with no endotamponade, while C3F8 gas and air were more frequently used in the PPVCE group.

**Table 3:** Types of Endotamponades Used in PPV and PPVCE Groups PPV: Pars plana vitrectomy; PPVCE: Pars plana vitrectomy with cataract extraction

Endotamponades	PPV (n=43)	PPVCE (n=72)
Perfluoropropane (C₃F₈) gas	12 (27.9%)	25 (34.7%)
Silicone oil	17 (39.5%)	29 (40.3%)
Air	6 (14.0%)	17 (23.6%)
None	8 (18.6%)	1 (1.4%)

Visual outcomes

In the PPV group, mean visual acuity improved significantly from preoperative to six months postoperatively, with further improvement noted at the last visit (p<0.01). The PPVCE group also demonstrated improvement, but the comparison between the two groups revealed no significant difference in visual outcomes (p=0.44) (Table 4). This indicates that while both procedures led to visual gains, the degree of improvement was comparable between the two groups.

**Table 4:** Comparison of Visual Outcomes Between PPV and PPVCE Post-Operation Two eyes in PPV group and eight eyes in PPVCE groups with PL or NPL vision were excluded from the visual acuity analysis. For the PPV group, both eyes were classified as PL or NPL vision post-operatively. In the PPVCE group, four eyes had PL vision pre-operatively; of these, two eyes remained PL/NPL post-operatively, while four eyes were classified as NPL post-operatively. PPV: Pars plana vitrectomy; PPVCE: Pars plana vitrectomy with cataract extraction; PL: perception to light; NPL: No perception to light Data presented as mean ± standard deviation (SD). Within-group comparisons (pre-operative vs post-operative BCVA) were performed using the paired t-test.

Procedure	N	Pre-operative logMAR	6 months Post-operative logMAR	logMAR at last visit	p-value (pre vs post-operation)
PPV	41	1.60±0.88	1.05±0.87	1.00±0.81	<0.01
PPVCE	64	1.58±0.87	1.21±0.88	1.13±0.90	

Visual outcomes by surgical indication

Visual outcomes post-operatively varied according to the surgical indication. For tractional retinal detachment (TRD), the PPV group's visual acuity post-operatively was slightly poorer, while the PPVCE group also showed a minor decline, with no significant difference between the two (p=0.54). In cases of vitreous hemorrhage (VH), both groups demonstrated significant improvements in visual acuity post-operatively (p<0.01). For patients with both TRD and VH, significant improvements were also observed in both groups post-operatively (p<0.01) (Table 5).

**Table 5:** Visual Outcomes by Surgical Indication *In the PPV TRD group, one eye had PL vision at the final visit. In the PPVCE TRD group, one eye had pre-operative PL vision, and two eyes had NPL vision at six months post-op. ^†^In the PPV VH group, one eye had NPL vision at six months. In the PPVCE VH group, one eye had pre-operative PL vision. ^‡^In the PPVCE TRD+VH group, two eyes had PL vision pre-operatively, one had NPL vision at six months and one had NPL at last visit. These eyes were excluded from the visual acuity analysis. PPV: Pars plana vitrectomy; PPVCE: Pars plana vitrectomy with cataract extraction; PL: perception to light; NPL: No perception to light; TRD: Tractional retinal detachment; VH: Vitreous hemorrhage Data are presented as mean ± standard deviation (SD). Within-group comparisons (pre-operative vs post-operative BCVA) were performed using the paired t-test.

Indication	Procedure	N	Pre-operative logMAR	6 months Post-operative logMAR	logMAR at last visit	p-value (pre vs post-operation)
TRD	PPV	15	1.15±0.55	1.24±0.89	1.24±0.87	0.540
	PPVCE*	34	1.29±0.72	1.40±1.00	1.33±1.03	
VH	PPV^†^	8	1.84±1.10	0.90±1.04	0.89±1.05	<0.01
	PPVCE	7	2.26 ±1.02	0.60±0.36	0.52±1.98	
TRD + VH	PPV	18	1.87±0.91	0.95±0.79	0.84±0.62	<0.01
	PPVCE^‡^	23	1.79±0.88	1.10±0.69	1.03±0.72	

Post-operative cataract extraction rates in the PPV group

In the PPV alone group, 48.8% of patients underwent cataract extraction after PPV during the study period, while 51.2% did not.

**Figure 1:**
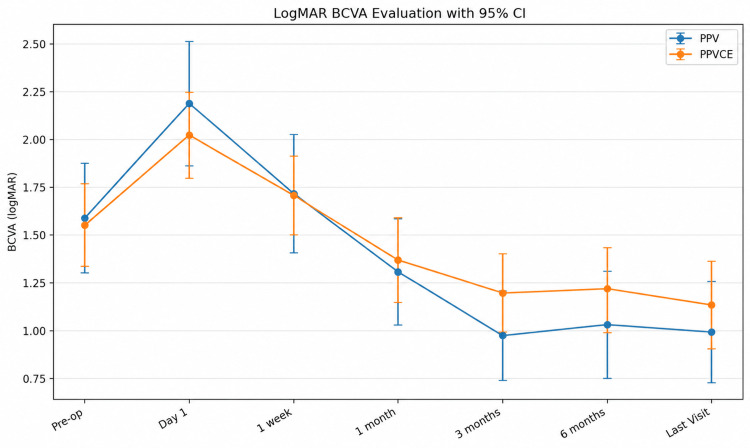
Visual acuity outcomes over time for PPV and PPVCE groups PPV: Pars plana vitrectomy; PPVCE: Pars plana vitrectomy with cataract extraction; BCVA: Best-corrected visual acuity; CI: Confidence interval

Both the PPV and PPVCE groups showed a decline in BCVA on post-operative day one, which is expected due to surgical trauma and inflammation, followed by a gradual improvement over time. At the six-month follow-up, the PPV group experienced a slight decrease in BCVA, likely due to cataract formation, which later improved, possibly reflecting the benefits of subsequent cataract extraction. In contrast, the PPVCE group showed continuous improvement without significant drops in visual acuity (Figure 1).

Post-operative outcomes

Post-operative ocular complications varied between the PPV and PPVCE groups, but no significant differences were observed in the rates of ocular hypertension, neovascular glaucoma, recurrent TRD, recurrent VH, fibrin exudation, or endophthalmitis. Ocular hypertension and neovascular glaucoma were more frequent in the PPVCE group, while endophthalmitis was reported in one patient from the PPV group (Table 6).

**Table 6:** Post-operative Ocular Complications in PPV and PPVCE Groups *Defined as ocular hypertension with an intraocular pressure (IOP) greater than 25 mmHg, requiring medication for management. PPV: Pars plana vitrectomy; PPVCE: Pars plana vitrectomy with cataract extraction; TRD: Tractional retinal detachment; VH: Vitreous hemorrhage

Complications	PPV (n=43)	PPVCE (n=72)
Ocular hypertension*	15 (34.9%)	33 (45.8%)
Neovascular glaucoma	3 (7.0%)	8 (11.1%)
Recurrent TRD	3 (7.0%)	6 (8.3%)
Recurrent VH	6 (14.0%)	6 (8.3%)
Fibrin exudation	0	3 (4.2%)
Endophthalmitis	1 (2.3%)	0

## Discussion

This study evaluates and compares the visual and post-operative outcomes of PPV and combined PPV with phacoemulsification (PPVCE) in diabetic patients with PDR and cataracts. The results evaluate visual outcomes according to indications for surgery and complication rates between the two types of surgeries.

In our study, a significant difference in age was observed between the PPV and PPVCE groups, with the PPVCE group being older (mean: 49.22 years) compared to the PPV group (mean: 43.30 years, p=0.001). This age disparity may be attributed to the higher prevalence of cataract formation in older patients, which necessitates concurrent cataract extraction during vitrectomy. Older individuals with diabetic retinopathy are more prone to cataract development due to cumulative oxidative stress and metabolic changes [11]. This aligns with previous studies reporting older age in patients undergoing combined surgery [8,10,12].

In comparing surgical indications between the PPV and PPVCE groups, our findings are consistent with Ben et al. [12], who similarly reported that TRD was the most common indication. In our study, TRD was more frequently observed in the PPVCE group, which may reflect a tendency to perform combined surgery in eyes with more advanced disease or where lens opacity may limit visualization or post-operative recovery. PPVCE may also be indicated where concurrent use of silicone oil is indicated, especially in cases of complicated TRD [12].

Both PPV and PPVCE resulted in significant improvements in visual acuity over time. Although pre-operative visual acuity was comparable between the two groups, no significant differences in post-operative visual outcomes were observed between PPV and PPVCE at the six-month and final follow-up visits. This is consistent with previous studies suggesting that while both procedures are effective in improving vision, the addition of phacoemulsification does not necessarily lead to superior visual outcomes compared to vitrectomy alone [9,10,12]. The comparable gains likely reflect effective management of underlying pathologies such as TRD and VH [3].

Interestingly, in the analysis of visual outcomes by surgical indication, both groups showed a marked improvement in patients with VH or TRD and VH. However, TRD alone showed limited visual recovery, likely due to irreversible retinal damage from ischemia, neurodegeneration, or structural changes [13].

A key finding of this study is the high rate of subsequent cataract surgery in the PPV-alone group, with 48.8% of patients requiring cataract extraction during follow-up. This highlights the clinical burden of staged procedures and the potential advantage of combined surgery in reducing the need for additional interventions. In contrast, PPVCE avoided this complication by removing the lens during vitrectomy, leading to a more stable visual acuity earlier on. This supports the advantage of combined surgery in patients with pre-existing lens opacities. However, combined surgery should be considered cautiously, particularly in younger patients with preserved accommodation and mild cataracts. It increases surgical complexity and recovery time. A poorly performed phacoemulsification may compromise the quality of the PPV by limiting the surgeon’s ability to clearly visualize and access the posterior segment for complete vitreous removal, membrane peeling, and hemostasis, ultimately affecting outcomes. Additionally, IOL-related issues such as edge glare or dysphotopsia should be taken into account when planning combined procedures [9].

Post-operative complication rates were comparable between the two groups. Although neovascular glaucoma (11.1% in PPVCE vs. 7.0% in PPV) and ocular hypertension (45.8% in PPVCE and 34.9% in PPV) were more frequent in the PPVCE group, these differences were not statistically significant. Some studies have suggested a higher risk of complications with combined surgery due to increased intraoperative manipulation and inflammation, but our findings align with others in demonstrating comparable safety profiles [10,12]. Overall, both PPV and PPVCE appear to be safe and effective surgical options for managing PDR with coexisting cataract.

The strengths of this study include a relatively large sample size and consistency in surgical technique, as all procedures were performed by the same vitreoretinal surgeon, thereby reducing inter-surgeon variability. However, this may limit generalizability across different surgeons and practice settings. Several limitations should be acknowledged. First, the retrospective design may be subject to selection bias and unmeasured confounding factors. Second, cataract morphology and severity (e.g., nuclear, cortical, or posterior subcapsular changes) were not uniformly graded using a standardized classification system, which may have influenced surgical selection and limited between-group comparability. Third, baseline differences between groups, particularly the older age of the PPVCE group and possible differences in cataract burden, were not adjusted using multivariate regression or propensity-based methods and may have influenced both treatment selection and post-operative outcomes. Therefore, between-group comparisons should be interpreted cautiously. Fourth, exclusion of eyes with PL or NPL vision from quantitative BCVA analysis may have biased visual results toward more favorable outcomes. Finally, although follow-up duration was adequate for short- to medium-term assessment, it may not fully capture late post-operative complications such as delayed neovascular glaucoma, recurrent vitreous hemorrhage, or recurrent retinal detachment.

## Conclusions

Both PPV and PPVCE achieve comparable visual and safety outcomes in patients with PDR and cataracts. However, the high incidence of subsequent cataract surgery following PPV alone highlights a consistent need for additional intervention, which may be mitigated by a combined approach. These findings suggest that PPVCE may help reduce the need for re-operations and improve overall treatment efficiency, particularly in patients with coexisting or anticipated cataract progression. Larger prospective comparative studies are warranted to further validate these findings.
